# When words count: the difference between recruitment manoeuvres and sigh

**DOI:** 10.1186/s40635-026-00884-7

**Published:** 2026-04-15

**Authors:** Anna Bortolaso, Marco Giani, Emanuele Rezoagli, Giuseppe Foti

**Affiliations:** Department of Medicine and Surgery, University of Milano-Bicocca, Via Cadore 48, 20900 Monza, Italy

Dear Editor,

We read carefully the recent article by Chiumello et al. [1] comparing two recruitment manoeuvre (RM) patterns in ARDS patients, defined as sustained inflation and “sigh recruitment”. While the study is methodologically rigorous, we believe that the terminology used deserves careful reconsideration. The intervention labelled as “sigh” does not correspond to its proper established physiological and clinical definition. This distinction is not semantic but methodological, and this misclassification may have implications for future evidence synthesis.

In respiratory physiology, a sigh is primarily a physiological phenomenon. It consists of augmented breaths typically two- to three-fold larger the average tidal volume and occurring at a low frequency, approximately 9/10 per hour. During spontaneous breathing, sighs occur intermittently [2] to maintain lung volume, prevent atelectasis and promote surfactant distribution. In mechanical ventilation, the concept has been translated into the intermittent delivery of pressure-controlled augmented breaths integrated within ongoing protective ventilation. The clinical effects of this manoeuvre have been evaluated in randomized trials. In the PROTECTION trial [3], sighs were administered over 3 s once per minute, achieving plateau pressures of 30 cmH_2_O. In the SiVent trial [4], they were delivered over 5 s every 6 min at 35/40 cmH_2_O of plateau pressures. Consistent with their physiological rationale, these studies reported safety and favorable physiological effects.

In the study by Chiumello et al., the manoeuvre labelled as “sigh recruitment” consisted of one minute of pressure-controlled ventilation with PEEP 5 cmH_2_O and a driving pressure of 40 cmH_2_O, resulting in plateau pressures of 45 cmH_2_O at 10 breaths per minute. Delivered as a sustained high–driving-pressure exposure, it lacks the defining features of a sigh—namely brevity, cyclicity, and integration within ongoing ventilation. Rather, it resembles a brief high-pressure recruitment manoeuvre, conceptually closer to repeated sustained inflations than to a cyclic augmented breath. As such, it differs substantially from the conventional understanding of a sigh.

The 2023 ESICM ARDS guidelines [5] clearly differentiate recruitment strategies according to pressure profile and duration. This distinction reflects differences in physiological rationale, available evidence, and resulting recommendations. Prolonged high-pressure manoeuvres (≥ 35 cmH_2_O for at least one minute) are recommended against; brief high-pressure manoeuvres are suggested against. In contrast, the guidelines describe periodic “sigh” breaths—performed one or more times every few minutes—as a distinct strategy intended to prevent progressive derecruitment during low tidal volume ventilation. However, they also emphasize that the potential benefits, optimal frequency, safety profile, and appropriate patient selection for cyclic sigh remain uncertain and warrant further investigation. Similarly, the 2024 ATS guideline recommends against prolonged recruitment manoeuvres while acknowledging uncertainty regarding shorter or cyclic strategies.

Using the same “sigh” label for physiologically distinct interventions risks obscuring these differences and may compromise valid comparisons across studies, with potential implications for future evidence synthesis and guideline development.

Greater consistency in terminology would help preserve conceptual clarity and ensure methodological coherence within the evolving ARDS literature.

## Data Availability

Not applicable.
